# Premature Decline of Serum Total Testosterone in HIV-Infected Men in the HAART-Era

**DOI:** 10.1371/journal.pone.0028512

**Published:** 2011-12-09

**Authors:** Vincenzo Rochira, Lucia Zirilli, Gabriella Orlando, Daniele Santi, Giulia Brigante, Chiara Diazzi, Federica Carli, Cesare Carani, Giovanni Guaraldi

**Affiliations:** 1 Chair and Unit of Endocrinology & Metabolism, Department of Medicine, Endocrinology & Metabolism, Geriatrics, University of Modena & Reggio Emilia, Azienda AUSL-NOCSAE of Baggiovara, Modena, Italy; 2 Metabolic Clinic, Infectious and Tropical Disease Unit, Department of Medicine and Medical Specialties, University of Modena & Reggio Emilia, Modena, Italy; South Texas Veterans Health Care System, United States of America

## Abstract

**Background:**

Testosterone (T) deficiency remains a poorly understood issue in men with Human Immunodeficiency Virus (HIV). We investigated the gonadal status in HIV-infected men in order to characterize T deficiency and to identify predictive factors for low serum T.

**Methodology/Principal Findings:**

We performed a cross-sectional, observational study on 1325 consecutive HIV male outpatients, most of them having lipodystrophy. Serum total T<300 ng/dL was used as the threshold for biochemical T deficiency. Morning serum total T, luteinizing hormone (LH), estradiol, HIV parameters, and body composition parameters by CT-scan and Dual-Energy-X-ray-Absorptiometry were measured in each case. Sexual behavior was evaluated in a subset of 247 patients. T deficiency was found in 212 subjects, especially in the age range 40–59, but was frequent even in younger patients. T deficiency occurred mainly in association with low/normal serum LH. Adiposity was higher in subjects with T deficiency (p<0.0001) and both visceral adipose tissue and body mass index were the main negative predictors of serum total T. Osteoporosis and erectile dysfunction were present in a similar percentage in men with or without T deficiency.

**Conclusions/Significance:**

Premature decline of serum T is common (16%) among young/middle-aged HIV-infected men and is associated with inappropriately low/normal LH and increased visceral fat. T deficiency occurs at a young age and may be considered an element of the process of premature or accelerated aging known to be associated with HIV infection. The role of HIV and/or HIV infection treatments, as well as the role of the general health state on the gonadal axis, remains, in fact, to be elucidated. Due to the low specificity of signs and symptoms of hypogonadism in the context of HIV, caution is needed in the diagnosis of hypogonadism in HIV-infected men with biochemical low serum T levels.

## Introduction

Circulating testosterone (T) declines with advancing age [1] especially after the age of 65 years [1]–[3]. The prevalence of T deficiency in different studies varies from 13.8% [4] to 20%–25% [5], [6] and 38.7% [3], depending on different cut-off values of serum T and different age ranges.

Androgen deficiency is common among men with human immunodeficiency virus (HIV) infection treated with highly active antiretroviral therapy (HAART), ranging from 20 to 30% according to different studies [7], [8]. In the past, men with acquired immunodeficiency syndrome (AIDS) showed a high prevalence of hypogonadism (29%–50%), mainly caused by impaired testicular function due to both wasting syndrome and opportunistic infections [9]–[12]. The introduction of HAART changed the spectrum and modified the prevalence of several co-morbidities, including androgen deficiency [11], which seems to occur less frequently than in the past [9], [10]. Despite its frequent occurrence, the actual prevalence of T deficiency remains not well defined in HIV-infected patients, ranging from 3–7% [7], [12], [13], [14], [17] to 54–64% [15], [16] in the different reports. An impairment of the hypothalamic-pituitary axis plays a possible major role in determining T deficiency, as suggested by the common occurrence of low T in concomitance with inappropriately low or normal serum luteinizing hormone (LH) in HIV-infected patients [12], [17], [18]. However, the underlying causes and mechanisms remain unknown. Previous studies on T deficiency in HIV-infected men suffer from several limitations: i) the lack of of information on gonadotropins in most studies [7], [12], [13], ii) the small number of subjects enrolled [7], [12]–[17], iii) the possibility of inappropriate blood sampling [7], [13]–[16] e.g. not considering serum T diurnal variations [19], iv) the measurement of free T alone [7], [16] by inaccurate assays [20], v) the retrospective design only from record charts [14], and, finally, vi) the lack of studies with an endocrinological rather than infectivological focus. These limitations weaken the evidence regarding T deficiency in HIV-infected men. As a result, clinical evidence resulting in the form of guidelines [21] or expert-based opinions [8], [22] is scarce.

At present, clinical practice guidelines for androgen deficiency recognize that HIV-infected men could be at higher risk of low serum T and perhaps of developing overt hypogonadism and indicate that T replacement treatment may be useful as an adjunctive therapy in HIV-infected men with low serum T but only in those with concomitant weight loss [21]. In subjects without HIV T deficiency is usually defined by investigating the hypothalamic pituitary-testicular axis. Accordingly, it is possible to differentiate primary hypogonadism (testicular failure) from secondary hypogonadism (gonadotropin deficiency) by measuring gonadotropin serum levels [21], [22]. In addition, several factors such as obesity or a poor health status could affect the hypothalamic-pituitary-gonadal axis in men [4], [6], [21], [22]. However, a systematic evaluation of T deficiency occurring in HIV-infected patients is still lacking, and the only available data come from small subsets of patients. Given the high frequency of T deficiency in HIV-infected patients [7], [12]–[17], understanding the pathophysiological mechanism of hypogonadism in these men, who may be considered for androgen replacement treatment, is essential for a rational treatment.

At present, two measurements of serum total T obtained in the morning (at 8 AM) documenting a T value below the lower limit of the laboratory range are required to confirm the diagnosis of hypogonadism [21]. The accuracy of T measurement is fairly controversial due to several limitations of the methods employed, especially those used for serum free T. Direct measurement of free T with commercially available kits is, in fact, unreliable, and the calculation of serum free T using sex hormone binding globulin (SHBG) and total T should be preferred [20]. However, even this parameter remains of limited value in clinical practice due to the possible sum of errors of each assay [23]. Measurements of free T by equilibrium dialysis and total T by tandem mass spectrometry are regarded as the gold standard in terms of accuracy and precision [20], [21], but are still expensive and time-consuming to be routinely used in clinical practice [23]. For these reasons, the repeated measurement of morning serum total T remains the most practical tool in the clinic and is the approach most widely used by clinicians to rule out T deficiency in men [21], [22], [23].

Considering all the factors mentioned above, we estimated the prevalence of low serum total T in a large cohort of HIV-infected men, predominately men with lipodystrophy. In particular, we evaluated T deficiency by measuring not only circulating T [21], but also serum LH in order to use the combination of T and LH to provide a comprehensive information on the gonadal axis function [4]. Combining LH with serum total T improves understanding of the nature of T deficiency in the context of HIV; furthermore it may be of help in the selection of candidates for T replacement treatment and strict monitoring. Furthermore, considering the changes in body composition occurring in HIV-infected men [11], [12], [18] and the potential impact of adiposity on the gonadal axis, we investigated the relationships of serum T with body composition and parameters of HIV infection and gonadotropin secretion, with the aim of identifying possible predictive factors for low serum T.

## Methods

**Figure 1:**
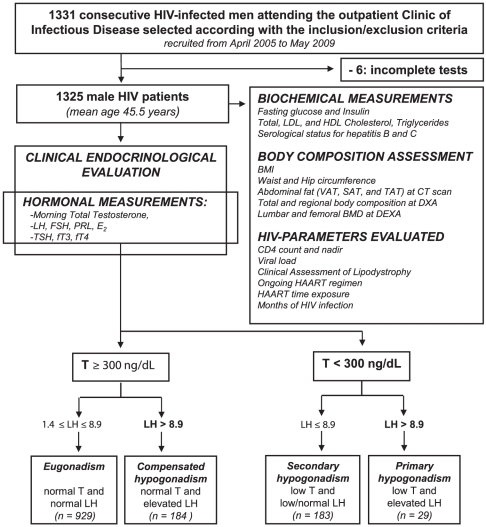
Study design. *E_2_: estradiol, PRL: prolactin, fT_4_: free T_4_, fT_3_: free T_3_*.

We performed a cross-sectional, observational study in HIV-infected patients attending the University Hospital of Modena as summarized in Figure 1.

### Participants

Fifteen hundred consecutive HIV-infected male outpatients who attended the Infectious Diseases Unit Metabolic Clinic for the assessment of lipodystrophy over a period from April 2005 to May 2009 were assessed for suitability before enrollment in the study. As lipodystrophy is very frequent among HIV-infected patients, body composition is currently assessed in patients with or without lipodystrophy in order to monitor prospectively body composition changes. 1331 HIV-infected men were finally enrolled in the study, according to the inclusion and exclusion criteria listed below. Six patients did not complete the protocol study and a total of 1325 patients aged 45.55±7.24 years underwent all the procedures planned by the study protocol (Figure 1).

### Study Description

All subjects underwent venous blood sampling at 8 AM after an overnight fast. All blood samples were stored at −80C° until assayed. The biochemical, endocrinological and anthropometric evaluations described below were performed in each participant enrolled according to the research protocol (Figure 1).

#### Inclusion criteria

Men aged 18–70 years, with serologically documented HIV infection, with or without lipodystrophy, and with ongoing HAART treatment. All patients were considered eligible if in a stable clinical condition without any acute, severe illness. The CD4 count was not considered as an inclusion criterium.

#### Exclusion criteria

Patients with prior treatment (referred or documented) with androgens, sex steroids, dehydroepiandrosterone, antiandrogens, anabolic agents, GnRH agonists, psycholeptic agents were considered not eligible.

Patients with documented hyperprolactinaemia (serum prolactin above 17.7 ng/mL), hypothyroidism, known pituitary, testicular or adrenal diseases, a previous conventional pituitary surgery or radiotherapy were considered not eligible.

A history of severe weight loss (more than 15% of the initial body weight), documented AIDS, cancer, previously documented diabetes mellitus, severe liver insufficiency or chronic renal failure, and a fasting glucose above 126 mg/dL, were considered as further exclusion criteria.

#### Hormonal and biochemical measurements

Serum LH, follicle stimulating hormone (FSH), prolactin, estradiol, total T, thyroid-stimulating hormone (TSH), free thyroxine (fT4), and free triiodothyronine (fT3) were measured.

Serological status for hepatitis B and C, total cholesterol, high-density lipoprotein (HDL), and low-density lipoprotein (LDL) cholesterol, triglycerides, glucose and insulin were assessed.

#### HIV infection-related parameters

The following parameters of HIV infection were evaluated: duration of HIV infection, quantitative plasma HIV RNA viral load, CD4 cell count, nadir CD4 count.

Information on both current HAART therapy and prior antiretroviral class use was obtained at the time of enrollment and duration of use for each drug was extracted from the medical records. The drugs were grouped according to the following antiretroviral classes: fusion inhibitors (FI), protease inhibitors (PI), non-nucleoside reverse transcriptase inhibitors (NNRTI), and nucleoside reverse transcriptase inhibitors (NRTI).

#### Anthropometric evaluation, body composition and adipose tissue distribution

Demographic characteristics (age, race, sex) and anthropometric measurements (weight, height, BMI) were recorded at the time of the first examination (Table 1). All patients underwent physical examination and lipodystrophy assessment. Lipodystrophy was defined according to the MACS (Multicenter AIDS Cohort Study) classification as “none”, “peripheral lipoatrophy”, “central adiposity” and “mixed form” [24]. Weight was measured after an overnight fast. Waist and hip circumferences were calculated as the average of 3 measurements. Height and weight were measured using standard calibrated instruments. Body mass index (BMI) was calculated as body weight (kilograms) divided by the square of height (square meters). Body composition and adipose tissue distribution were evaluated by both Dual-Energy-X-ray-Absorptiometry (DXA) and abdominal CT-scan, respectively. An abdominal CT-scan was performed in order to determine accurately visceral (VAT), subcutaneous (SAT) and total (TAT) abdominal adipose tissue. Whole body composition assessment (lean and fat mass) and measurement of bone mineral density (BMD) were both obtained by DXA (Hologic-QDR-2000 densitometer, Inc., Waltham, MA). This technique has an accuracy of ±3% for fat mass and ±1.5% for fat-free mass, respectively [25]. BMD was measured at the femoral neck and lumbar spine.

**Table 1:** Characteristics of the entire cohort as median, and minimum and maximum in parenthesis.

*n 1325*	n.v.	Median (Min-Max)
**Age (years)**	-	45 (20–69)
**Anthropometrical variables**		
Weight (kg)	-	70.5 (42–135)
BMI (kg/m^2^)	19–25	23.62 (15.01–54.97)
Waist circumference (cm)	<94	87 (63–160)
Hip circumference (cm)	<101	89.0 (71–141)
Waist/hip circumference ratio	<0.95	0.97 (0.79–1.29)
VAT (cm^2^)	-	129 (11–853)
SAT (cm^2^)	-	100 (2–807)
TAT(cm^2^)	-	237 (16–1088)
Total body fat mass (g·1.000)	-	9.19 (1.10–46.23)
Trunk fat mass (g·1.000)	-	5.63 (0.25–47.43)
Total body lean mass (g·1.000)	-	55.47 (4.57–84.19)
Trunk lean mass (g·1.000)	-	27.66 (2.45–45.43)
**Biochemical measurements**		
Total cholesterol (mg/dL)	<200	184 (66–390)
LDL cholesterol (mg/dL)	<115	108 (32–240)
HDL cholesterol (mg/dL)	>39	39 (15–80)
Triglycerides (mg/dL)	<180	184 (29–981)
Glucose (mg/dL)	70–110	91 (58–122)
Insulin (uU/mL)	2–23	14.4 (0.5–165)
**Hormonal measurements**		
Total T (ng/dL)	300–900	452 (11–1098)
LH (mIU/mL)	1.4–8.9	5 (0.1–46.2)
FSH (mIU/mL)	1.7–6.9	4.6 (0.3–75.6)
E2 (pg/mL)	20–40	31 (4.6–98)
Prolactin (ng/mL)	2.1–17.7	6.6 (2.2–17.5)
TSH (uU/mL)	0.35–4.5	1.72 (0.53–4.3)
fT4 (ng/dL)	0.61–1.67	1.04 (0.75–1.59)
fT3 (pg/mL)	1.7–4.2	3.4 (1.78–4.05)
**Bone Mineral Density**		
Lumbar BMD (g/cm^2^)	-	1.07 (0.65–1.76)
Lumbar normal BMD *n (%)*	-	*714 (53.90%)*
Lumbar osteopenic BMD *n (%)*	-	*494 (37.28%)*
Lumbar osteporosis BMD *n (%)*	-	*117 (8.82%)*
Femoral BMD (g/cm^2^)	-	0.85 (0.32–1.41)
Femoral normal BMD *n (%)*	-	*480 (36.26%)*
Femoral osteopenic BMD *n (%)*	-	*715 (53.93%)*
Femoral osteporosis BMD *n (%)*	-	*130 (9.81%)*
**Infectivological parameters**		
CD4 Count (cells/mm^3^)	>400	500 (11–2459)
CD4 nadir (cells/mm^3^)	-	170 (0–986)
Viral Load	-	50 (20–94988)
Suppressed viral load *n (%)*	<40 copies/mL	*901 (68%)*
Months of HIV infection	-	175 (3–285)
Hepatitis B *n (%)*	-	*78 (5.89%)*
Hepatitis C *n (%)*	-	*380 (28.68%)*
**Lipodystrophy – MACS score**		
None *n (%)*	-	*184 (13.90%)*
Peripheral lipoatrophy *n (%)*	-	*573 (43.20%)*
Central adiposity *n (%)*	-	*123 (9.30%)*
Mixed form *n (%)*	-	*445 (33.60%)*
**Ongoing therapy**		
FI *n (%)*	-	*21 (1.58%)*
PI *n (%)*	-	*447 (33.74%)*
NNRTI *n (%)*	-	*331 (24.98%)*
NRTI *n (%)*	-	*850 (64.15%)*
**Time exposure to HAART**		
FI (months)		8 (0–45)
PI (months)		25 (0–184)
NNRTI (months)		22 (0–118)
NRTI (months)		49 (0–262)
Total duration of HAART (years)		10 (1–16)

*Number of patients (n) and relative percentages (%) are reported in Italics, n.v.: normal values: E_2_: estradiol, fT_4_: free T_4_, fT_3_: free T_3_, BMD: bone mineral density, FI: fusion inhibitors, PI: protease inhibitors, NNRTI: non-nucleoside reverse transcriptase inhibitors, NRTI: nucleoside reverse transcriptase inhibitors.*

As per standard protocols, the abdominal adipose tissue distribution was studied by CT-scan at L4 level, with a single slice abdominal scan in order to determine VAT and SAT [26], TAT being the sum of VAT and SAT.

#### Signs and symptoms of hypogonadism

Subjects were considered as having a normal BMD, osteopenia, or osteoporosis when the t-score was ≥−1, between <−1 and >−2.5, and ≤−2.5, respectively [27].

Sexual function was studied in a subset of 247 subjects by means of the 15-item International Index of Erectile Function (IIEF-15) questionnaire [28], [29]. The IIEF-15 is a widely used, multidimensional self-report instrument for the evaluation of male sexual function [28]. Scientists recommend IIEF-15 as a primary instrument in clinical trials of erectile dysfunction (ED) and for the diagnostic evaluation of ED severity [29]. The IIEF-15 questionnaire addresses five domains of male sexual function: erectile function, orgasmic function, sexual desire, intercourse satisfaction, and overall satisfaction with sex life [28], [29]. All responses to the IIEF-15 questions are rated on a 5-point scale, with a score of 1 representing the worst and 5 the best response.

#### Criteria for the classification of the gonadal status

Total serum T was considered as the main marker of biochemical T deficiency and patients were classified as having normal or low serum total T according to the threshold value of 300 ng/dL (10.4 nmol/L) corresponding to the lowest limit of the normal range of total T assay in the Laboratory of Modena [21] (Figure 1). According to Tajar et al. [4], serum LH was used to assign patients to the following categories of biochemical hypogonadism : 1) eugonadism (normal T and normal LH), 2) compensated hypogonadism (normal T and high LH), 3) secondary hypogonadism (low T and low or normal LH), and 4) primary hypogonadism (low T and high LH) (Figure 1).

#### Laboratory analysis

Serum total T was assayed by immunochemioluminescence (ADVIA Centaur, Siemens). The inter- and intra-assay coefficients of variation were 7.1% and 4.8%, respectively.

Serum LH, FSH, and prolactin were assayed by immunochemioluminescence (ADVIA Centaur, Siemens). The inter- and intra-assay coefficients of variation were of 3.6% and 2.4% for LH, 5.2% and 2.6% for FSH, 3.9% and 2.5% for prolactin.

Serum estradiol (E_2_) was assayed by RIA (Third-Generation DSL-39100, Diagnostic Systems Laboratories, Inc., Webster, TX, USA) with a sensitivity of 0.6 pg/mL (2.2 pmol/L), the inter- and intra-assay coefficients of variation were between 4.1 and 9.9%, and between 3.4 and 3.9%, respectively.

TSH, fT3, fT4, LDL-, HDL- and total cholesterol, triglycerides, glucose and insulin were assayed using commercially available kits. The CD4 cell count was performed by flow cytometry (Becton Dickinson Immunocytochemistry Systems, San Jose, CA). Quantitative plasma HIV RNA viral load was performed using an ultrasensitive method (Amplicor HIV-1 Monitor Assay, Roche Molecular Systems, Indianapolis, IN).

### Ethics

The study received ethical approval from the Institutional Review Board of Modena (Comitato Etico di Modena) and all participants in the study provided written informed consent.

### Statistical methods

The nonparametric Mann-Whitney test was used for comparisons since most of the variables resulted being not normally distributed by the Kolmogorov-Smirnov test. The comparison among groups was performed using the nonparametric Kruskal-Wallis test (when more than two groups) followed by the Dunn's multiple comparison post hoc test when a significant difference was found in order to establish differences between individual groups. All the data are shown as median and minimum-maximum.

We evaluated demographic, anthropometric and hormonal variables in order to identify possible predictive factors for circulating serum T. A stepwise, linear, multiple regression analysis was performed using serum T as the dependent variable, serum LH as the forced entry variable, followed by age, BMI, waist circumference, hip circumference, VAT, SAT, TAT, total body fat, truncal fat, total lean mass and truncal lean mass at DXA, and HIV infection parameters (months of HIV-infection, viral load, CD4 count and CD4 nadir) as independent variables. All multiple regression analyses were based on a single regression analysis of each predictive independent variable that allowed identification of candidate predictive variables. A univariate analysis was performed prior to multivariate analysis. Data are not shown when the results were not significant. The independent variables with a significance of p<0.05 were entered into the regression model. Stepwise, linear multiple regression analysis using a backwards elimination method was applied to the data, with p<0.10 as the criterion for a variable to be considered in the model. The percentage of contribution of a given variable to the variance of serum T was determined by using the square of the Pearson correlation coefficient (r^2^).

Statistical analysis was performed using the ‘Statistical Package for the Social Sciences’ software for Windows (version 16.0; SPSS Inc., Chicago, IL) and Sigma Plot (version 11.00 for Windows; Systat Software Inc., San Jose, CA) for Kruskal-Wallis and the Dunn's tests. For all comparisons, a p value<0.05 was considered statistically significant.

## Results

The clinical characteristics of the 1325 patients studied and information concerning the HIV status of the whole cohort are summarized in Table 1. According to the MACS score 13.9% of the enrolled patients were not affected by lipodystrophy, the remaining subjects being all lipodystrophic (Table 1).

All parameters in the patients, grouped according to serum T levels, are reported in Table 2 and 3. All parameters in the patients grouped according to both serum T and LH levels are reported in Table 3.

**Table 2:** Characteristics as median, and minimum and maximum in parenthesis, according to the two groups defined by using the threshold of serum total T of 300 ng/dL for establishing T deficiency (the nonparametric Mann-Whitney test was used for comparisons).

	n.v.	Total T≥300 ng/dL	Total T<300 ng/dL	Sig.
***n 1325 (%)***		*1113 (84%)*	*212 (16%)*	
**Age (years)**	-	45 (20–69)	46 (28–68)	p = 0.006
**Anthropometrical variables**				
BMI (kg/m^2^)	19–25	23.37 (15.01–43.49)	25.13 (17.2–54.97)	p<0.0001
Waist circumference (cm)	<94	86 (63–128)	90 (68–160)	p<0.0001
Hip circumference (cm)	<101	89 (71–116)	91 (77.5–141)	p<0.0001
Waist/hip circumference ratio	<0.95	0.97 (0.79–1.29)	0.99 (0.84–1.21)	p<0.0001
VAT (cm^2^)	-	124 (11–853)	151 (29–521)	p<0.0001
SAT (cm^2^)	-	97 (2–807)	124 (2–595)	p<0.0001
TAT(cm^2^)	-	230 (16–1088)	288 (56–909)	p<0.0001
Total body fat mass (g·1.000)	-	8.91 (1.10–46.23)	11.7 (1.95–43.42)	p<0.0001
Trunk fat mass (g·1.000)	-	5.43 (0.25–47.43)	6.98 (1.0–27.87)	p<0.0001
Total body lean mass (g·1.000)	-	55.23 (4.57–84.19)	57.34 (5.43–83.93)	p = 0.002
Trunk lean mass (g·1.000)	-	27.49 (2.45–43.32)	28.63 (6.35–45.43)	p = 0.002
**Hormonal measurements**				
Total T (ng/dL)	300–900	488 (300–1098)	247 (11–299)	p<0.0001
LH (mIU/mL)	1.4–8.9	5.3 (1.4–46.2)	3.8 (0.1–40.8)	p<0.0001
FSH (mIU/mL)	1.7–6.9	4.6 (0.3–75.6)	4.5 (0.3–42.7)	p = 0.6
E2 (pg/mL)	20–40	32 (4.6–98)	23 (10–91)	p<0.0001
E2/Total T	-	0.0066 (0.0008–0.0219)	0.0101 (0.0036–0.8)	p<0.0001
**Bone Mineral Density**				
Lumbar BMD (g/cm^2^)	-	1.08 (0.65–1.76)	1.07 (0.70–1.66)	p = 0.7
Lumbar normal BMD *n (%)*	-	*603 (54,17%)*	*112 (52.84%)*	-
Lumbar osteopenic BMD *n (%)*	-	*411 (36,93%)*	*83 (39.16%)*	-
Lumbar osteporosis BMD *n (%)*	-	*99 (8,9%)*	*17 (8.0%)*	-
Femoral BMD (g/cm^2^)	-	0.85 (0.32–1.41)	0.86 (0.44–1.29)	p = 0.5
Femoral normal BMD *n (%)*	-	*402 (36.12%)*	*78 (36.8%)*	-
Femoral osteopenic BMD *n (%)*	-	*597 (53.64%)*	*117 (55.2%)*	-
Femoral osteporosis BMD *n (%)*	-	*114 (10.24%)*	*17 (8.0%)*	-
**Infectivological parameters**				
CD4 Count (cells/mm^3^)	>400	495.5 (11–1670)	525 (11–2459)	p = 0.1
CD4 nadir (cells/mm^3^)	-	166 (0–870)	184.50 (1–986)	p = 0.2
Viral Load	-	50 (20–94988)	50 (20–93265)	p = 0.9
Months of HIV infection	-	177 (3–285)	165 (10–285)	p = 0.7
**Lipodystrophy – MACS score**				
None *n (%)*	-	*162 (14.55%)*	*22 (10.38%)*	-
Periferal lipoatrophy *n (%)*	-	*500 (44.92%)*	*72 (33.96%)*	-
Central adiposity *n (%)*	-	*91 (8.18%)*	*33 (15.57%)*	-
Mixed form *n (%)*	-	*360 (32.35%)*	*85 (40.09%)*	-
**Ongoing therapy**				
FI *n (%)*	-	*17 (1,53%)*	*4 (1.89%)*	-
PI *n (%)*	-	*386 (34.68%)*	*61 (28.77%)*	-
NNRTI *n (%)*	-	*261 (23.45%)*	*70 (33.02%)*	-
NRTI *n (%)*	-	*704 (63.25%)*	*146 (68.87%)*	-
**Time exposure to HAART**				
FI (months)	-	8 (0–45)	6 (1–18)	p = 0.7
PI (months)	-	27 (0–184)	18.50 (0–164)	p = 0.047
NNRTI (months)	-	24 (0–118)	17.5 (0–108)	p = 0.2
NRTI (months)	-	53.50 (0–262)	18.50 (0–218)	p = 0.004
Total duration of HAART (years)		10 (1–16)	10 (1–14)	p = 0.7

*Number of patients (n) and relative percentages (%) are reported in Italics, n.v.: normal values, E_2_: estradiol, BMD: bone mineral density, FI: fusion inhibitors, PI: protease inhibitors, NNRTI: non-nucleoside reverse transcriptase inhibitors, NRTI: nucleoside reverse transcriptase inhibitors.*

**Table 3:** Number of patients (*n*) and relative percentages (*%*) of patients according to age and total T serum levels.

Age (years)	Entire cohort	Total T≥300 ng/dL	Total T<300 ng/dL
20–29 n (%)	12 (0.9%)	11 (91.7%)	1 (8.3%)
30–39 n (%)	207 (15.6%)	185 (89.4%)	22 (10.6%)
40–49 n (%)	800 (60.4%)	677 (84.7%)	123 (15.3%)
50–59 n (%)	245 (18.5%)	187 (76.4%)	58 (23.6%)
60–69 n (%)	61(4.6%)	53 (86.9%)	8 (13.1%)

### Prevalence of biochemical T deficiency and Categories of Hypogonadism

Total serum T levels were below 300 ng/dL (10.4 nmol/L) in 212 (16%) subjects, with the highest rate of low serum total T in men aged 40–49 and 50–59. Remarkably, a relevant percentage of men with impaired total serum T (10.6%) was found in patients aged 30–39 (Table 3).

Considering both serum total T and LH levels, the patients were grouped according to their gonadal status in four categories : a) eugonadism with normal serum T≥300 ng/dL and LH (1.4≤LH≤8.9 mIU/mL): 929 subjects (70%); b) compensated hypogonadism with normal serum T≥300 ng/dL and elevated LH (>8.9 mIU/mL): 184 subjects (14%); c) secondary hypogonadism with low serum T<300 ng/dL and low (<1.4 mIU/mL) to normal (1.4≤LH≤8.9 mIU/mL) serum LH: 183 subjects (14%); and d) primary hypogonadism with low serum T<300 ng/dL and elevated LH (>8.9 mIU/mL): 29 (2%) subjects (Table 4).

**Table 4:** Characteristics as median, and minimum and maximum in parenthesis, according to the serum total T threshold of 300 ng/dL in tandem with the LH serum levels (the comparison among groups was performed by nonparametric Kruskal-Wallis test followed by the Dunn's multiple-comparison post hoc test when a significant difference was found in order to establish differences among each group).

	Total T≥300 ng/dL		Total T<300 ng/dL		Sig.
*n 1325 (%)*	*1113 (84%)*		*212 (16%)*		
	E	CH	SH	PH	
***n 1325 (%)***	*929 (70%)*	*184 (14%)*	*183 (14%)*	*29 (2%)*	-
Age (years)	45 (20–69)	45 (30–69)	45 (28–68)	48 (38–66)	p = 0.005
**Anthropometrical variables**					
BMI (kg/m^2^)	23.5 (15.4–43.5)	22.5 (15–32.8)	25.2 (17.2–54.97)	24 (17.6–35.9)	p<0.0001
Waist circumference (cm)	86.5 (68–128)	85.5 (63–116)	90 (68–160)	87 (74–119)	p<0.0001
Hip circumference	89 (76–116)	88 (71–104)	91 (77.5–141)	90 (79–102)	p<0.0001
Waist/hip circumference ratio	0.97 (0.79–1.29)	0.96 (0.82–1.2)	0.99 (0.84–1.2)	0.99 (0.9–1.21)	p<0.0001
VAT (cm^2^)	129 (11–853)	109 (14–446)	151 (29–521)	149 (42–369)	p<0.0001
SAT (cm^2^)	99 (2–807)	76.5 (2–747)	125 (2–595)	118 (10–398)	p<0.0001
TAT(cm^2^)	237 (16–1088)	211 (16–894)	287 (56–909)	292 (16–630)	p<0.0001
Total body fat mass (g·1.000)	9.1 (1.1–46.2)	8.3 (1.7–25.3)	11.7 (1.9–43.4)	9.3 (2.9–27.4)	p<0.0001
Trunk fat mass- (g·1.000)	5.6 (0.25–47.4)	4.8 (0.93–25.8)	7.0 (1.0–26.6)	6.0 (1.6–27.8)	p<0.0001
Total body lean mass- (g·1.000)	55.4 (4.6–82.2)	53.8 (33.5–84.19)	58 (5.4–84.0)	53.3 (41.0–69.7)	p<0.0001
Trunk lean mass- (g·1.000)	27.5 (2.4–43.3)	27.2 (4.7–42.7)	28.7 (6.3–45.4)	26.8 (6.6–39.0)	p<0.0001
**Bone Mineral Density**					
Lumbar BMD (g/cm^2^)	1.07 (0.65–1.76)	1.08 (0.65–1.60)	1.07 (0.70–1.66)	1.09 (0.88–1.44)	p = 0.9
Lumbar normal BMD *n (%)*	*504 (54.30%)*	*98 (53.33%)*	*95 (51.91%)*	*17 (58.62%)*	-
Lumbar osteopenic BMD *n (%)*	*346 (37.20%)*	*66 (35.83%)*	*73 (39.89%)*	*10 (34.48%)*	-
Lumbar osteporosis BMD *n (%)*	*79 (8.50%)*	*20 (10.84%)*	*15 (8.2%)*	*2 (6.90%)*	-
Femoral BMD (g/cm^2^)	0.85 (0.32–1.41)	0.85 (0.46–1.29)	0.86 (0.44–1.29)	0.86 (0.63–1.20)	p = 0.9
Femoral normal BMD *n (%)*	*341 (36.70%)*	*73 (39.67%)*	*67 (36.61%)*	*11 (37.93%)*	-
Femoral osteopenic BMD *n (%)*	*503 (54.15%)*	*85 (46.19%)*	*102 (55.73%)*	*15 (51.72%)*	-
Femoral osteporosis BMD *n (%)*	*85 (9.15%)*	*26 (14.14%)*	*14 (7.66%)*	*3 (10.35%)*	-
**Hormonal measurements**					
Total T (ng/dL)	478 (300–1098)	553.5 (303–1097)	247 (11–299)	235 (94–299)	p<0.0001
LH (mIU/mL)	4.7 (1.4–8.9)	11.65 (9.1–46.2)	3.5 (0.1–8.9)	11.5 (9–40.8)	p<0.0001
FSH (mIU/mL)	4.2 (0.3–29.8)	9 (1.4–75.6)	4.2 (0.3–15.7)	14.2 (1.4–42.7)	p<0.0001
E2 (pg/mL)	32 (4.6–98)	37 (10–97)	23.5 (10–91)	23 (10–52)	p<0.0001
E2/Total T	0.0066 (0.0008–0.022)	0.006 (0.0018–0.018)	0.0101 (0.0036–0.8)	0.0100 (0.0041–0.031)	p<0.0001
**Infectivological parameters**					
CD4 Count (cells/mm^3^)	504 (11–1670)	459 (11–1261)	547.5 (11–2459)	421.5 (34–1320)	p<0.0001
CD4 nadir (cells/mm^3^)	180 (0–870)	110.50 (0–629)	200 (1–986)	115 (4–500)	p<0.0001
Viral Load	50 (20–94988)	50 (40–94875)	50 (20–93265)	50 (40–91458)	p = 0.5
Months of HIV infection	172 (3–285)	215 (29–279)	162 (10–280)	190 (73–285)	p<0.0001
**Lipodystrophy – MACS score**					
None *n (%)*	*148 (15.93%)*	*14 (7.6%)*	*21 (11.44%)*	*1 (3.45%)*	-
Periferal lipoatrophy *n (%)*	*409 (44.03%)*	*91 (49.46%)*	*59 (32.30%)*	*13 (44.82%)*	-
Central adiposity *n (%)*	*80 (8.61%)*	*11 (5.98%)*	*29 (15.84%)*	*4 (13.79%)*	-
Mixed form *n (%)*	*292 (31.43%)*	*68 (36.96%)*	*74 (40.42%)*	*11 (37.94%)*	-
**Ongoing therapy**					
FI *n (%)*	*15 (1.61%)*	*2 (1.09%)*	*2 (1.09%)*	*2 (6.90%)*	-
PI *n (%)*	*302 (32.51%)*	*84 (45.65%)*	*53 (28,96%)*	*8 (27.59%)*	-
NNRTI *n (%)*	*223 (24.00%)*	*38 (20.65%)*	*57 (31.15%)*	*13 (44.83%)*	-
NRTI *n (%)*	*579 (62.32%)*	*125 (67.93%)*	*125 (68.31%)*	*21 (72.41%)*	-
**Time exposure to drugs**					
FI (months)	6.50 (0–40)	13 (2–45)	6 (1–18)	9 (6–12)	p = 0.055
PI (months)	27 (0–184)	29 (0–172)	18 (0–164)	36 (8–104)	p = 0.073
NNRTI (months)	24.50 (0–118)	16 (1–89)	17 (0–108)	31 (1–82)	p = 0.037
NRTI (months)	53.50 (0–262)	54.50 (1–225)	18 (0–218)	72.50 (1–180)	p = 0.009
	**NORMAL LH**	**IMPAIRED LH**			
***n 1325 (%)***	***929 (70%)***	***396 (30%)***			

*Number of patients (n) and relative percentages (%) are reported in Italics, E: Eugonadism (Normal T and Normal LH), CH: Compensated Hypogonadism (Normal T and Elevated LH), SH: Secondary Hypogonadism (Low T and Low to Normal LH), PH: Primary Hypogonadism (Low T and Elevated LH),, E_2_: estradiol, BMD: bone mineral density, FI: fusion inhibitors, PI: protease inhibitors, NNRTI: non-nucleoside reverse transcriptase inhibitors, NRTI: nucleoside reverse transcriptase inhibitors.*

**Figure 2:**
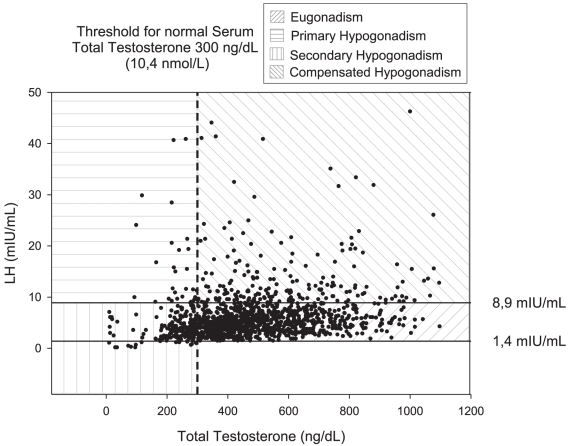
Gonadal status according to serum total T threshold of 300 ng/dL and LH normal range.

Based on both LH and total T serum levels the prevalence of biochemical hypogonadism, defined as serum T<300 ng/dL or an inappropriately high serum LH value with normal serum T, (comprising patients with compensated, secondary and primary hypogonadism) increased from 16% to 30% (Figure 2, Table 4).

### Comparison between groups according to serum total T

Variables including BMI, waist circumference, VAT, SAT, TAT, total body fat, and truncal fat (p<0.0001), total lean body mass and trunk lean body mass (p = 0.002), and age (p = 0.006) were all significantly higher in subjects with serum total T<300 ng/dL (<10.4 nmol/L) than in subjects with normal serum total T (Table 2). Serum LH and estradiol were significantly lower in subjects with serum total T<300 ng/dL than in subjects with normal serum total T (p<0.0001). No difference was found in the CD4 count, the CD4 nadir, the viral load, the number of months of HIV infection, and serum FSH (Table 2) between subjects with serum total T<300 ng/dL and those with normal serum total T. No substantial difference was found in the class of antiretroviral drugs used and in the time of exposure to the drugs (Table 2), except for the duration of exposure to PI (p<0.05), and NRTI (p<0.005), which resulted longer in patients with normal T than in patients with T deficiency (Table 2). The percentage of men having osteopenia or osteoporosis was unexpectedly similar between subjects with T deficiency and subjects with normal T. Furthermore, both femoral and lumbar BMD were not significantly different between the two groups (Table 2). The IIEF-15 score, measured in a subset of 247 subjects, often resulted below the threshold commonly used for defining the presence (<25) or the lack (≥25) of ED [28], [29]. This result is consistent with a remarkably high frequency of ED (53.44%) (Table 5). The mean score of erectile function was higher in men with normal serum T than in men with total serum T<300 ng/dL, but the percentage of men with an insufficient erectile function score remained elevated (49%) even within men with normal circulating T (Table 6). The remaining IIEF-15 domains (orgasmic function, sexual desire, intercourse satisfaction, and overall satisfaction with sex life) showed a mean below the lower limit of the normal range, with the percentage of abnormal scores always >50% (Table 5). All scores of these domains were significantly lower in subjects with serum T<300 ng/dL than in those with normal T, but the percentage of men with an insufficient score was elevated even in men with normal T (Table 6).

**Table 5:** Characteristics of the 247 patients who underwent International Index of Erectile Function (IIEF-15) questionnaire (the nonparametric Mann-Whitney test was used for comparisons since most of the variables resulted not normally distributed at Kolmogorov-Smirnov test).

n 247	n.v.	Median (Min-Max)
**Age (years)**	-	44 (30–69)
**Anthropometrical variables**		
Weight (kg)	-	71.0 (42–125)
BMI (kg/m^2^)	19–25	23.86 (15.3–40.35)
Waist circumference (cm)	<94	87 (70–128)
**Hormonal measurements**		
Total T (ng/dL)	300–900	442 (19.5–989)
LH (mIU/mL)	1.4–8.9	5 (1.1–31.8)
FSH (mIU/mL)	1.7–6.9	4.5 (0.3–48.3)
E2 (pg/mL)	20–40	31 (10–89)
Prolactin (ng/mL)	2.1–17.7	6.5 (2.4–17.3)
**IIEF-15 Parameters**		
Erectile function	≥25	24 (0–30)
Orgasmic function	≥9	8 (0–10)
Sexual desire	≥9	8 (0–10)
Intercourse satisfaction	≥10	9 (0–15)
Overall satisfaction	≥9	7 (0–10)
Erectile function <25 *n (%)*	-	*132 (53.44%)*
Orgasmic function <9 *n (%)*	-	*129 (52.23%)*
Sexual desire <9 *n (%)*	-	*161 (65.2%)*
Intercourse satisfaction <10 *n (%)*	-	*132 (53.44%)*
Overall satisfaction <9 *n (%)*	-	*210 (85.02%)*

*Number of patients (n) and relative percentages (%) are reported in Italics, n.v.: normal values; E_2_: estradiol. The erectile function is the domain used in clinical practice to rule out erectile dysfunction when a score of 25 or more is obtained* [*29*] *.*

**Table 6:** Characteristics of the 247 patients who filled the International Index of Erectile Function (IIEF-15) questionnaire according to the threshold of serum total T of 300 ng/dL used to define T deficiency (the nonparametric Mann-Whitney test was used for comparisons since most of the variables resulted not normally distributed at Kolmogorov-Smirnov test).

	n.v.	Total T≥300 ng/dL	Total T<300 ng/dL	Sig.
***n 247 (%)***		*220 (89%)*	*27 (11%)*	
**Age (years)**	-	45 (30–69)	44 (33–59)	p<0.05
**Anthropometrical variables**				
BMI (kg/m^2^)	19–25	23.7 (15.3–37.7)	25.5 (19.68–40.35)	p<0.005
Waist circumference (cm)	<94	87 (70–128)	90 (79–125.5)	p<0.005
**Hormonal measurements**				
Total T (ng/dL)	300–900	476 (300–989)	250 (19.5–297)	p<0.005
LH (mIU/mL)	1.4–8.9	5.25 (1.4–31.8)	3.7 (1.1–28.4)	p<0.005
**IIEF-15 Parameters**				
Erectile function	≥25	25 (0–30)	18 (1–29)	p = 0.003
Orgasmic function	≥9	8 (0–10)	6 (0–10)	p = 0.019
Sexual desire	≥9	8 (0–10)	7 (2–9)	p = 0.002
Intercourse satisfaction	≥10	9 (0–15)	8 (0–13)	p = 0.082
Overall satisfaction	≥9	7 (0–10)	5 (0–8)	p = 0.003
Erectile function <25 *n (%)*	-	*108 (49%)*	*20 (74.1%)*	-
Orgasmic function <9 *n (%)*	-	*111 (50.5%)*	*18 (66.7%)*	-
Sexual desire <9 *n (%)*	-	*138 (62.73%)*	*23 (85.2%)*	-
Intercourse satisfaction <10 *n (%)*	-	*115 (52.27%)*	*17 (63%)*	-
Overall satisfaction <9 *n (%)*	-	*183 (83.2%)*	*27 (100%)*	-

*Number of patients (n) and relative percentages (%) are reported in Italics, n.v.: normal values. The erectile function is the domain used in clinical practice to rule out erectile dysfunction when a score of 25 or more is obtained* [*29*] *.*

### Comparison among groups according to categories of hypogonadism

FSH, estradiol, the E_2_/T ratio, BMI, waist circumference, VAT, SAT, TAT, total body fat, truncal fat, total lean body mass, trunk lean body mass, CD4 count, CD4 nadir, and the number of months of HIV infection were all significantly different among the four groups of HIV-infected men designated as eugonadism, compensated hypogonadism, secondary hypogonadism, and primary hypogonadism (p<0.0001) (Table 4). Age was significantly different among the groups as well (p = 0.005) (Table 4).

**Figure 3:**
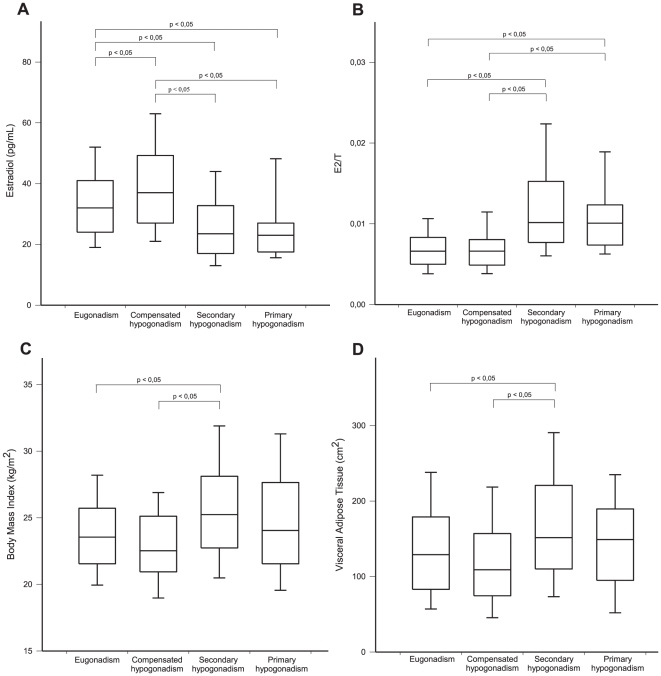
Comparison among groups according to categories of hypogonadism. Serum estradiol (E_2_), (a), E_2_/T ratio (b), BMI (c), and VAT (d) in HIV infected men with eugonadism (normal T and normal LH) compared to compensated hypogonadism (normal T and elevated LH), secondary hypogonadism (low T and low/normal LH), and primary hypogonadism (low T and elevated LH). *The line and the whiskers on the box plots represents median and Standard Errors, respectively*.

Serum estradiol was significantly higher in HIV-infected men with eugonadism and compensated hypogonadism compared with men with secondary or primary hypogonadism (p<0.05) (Figure 3a), but the E_2_/T ratio was significantly higher in men with secondary hypogonadism than in subjects with eugonadism and compensated hypogonadism (p<0.05) (Figure 3b). The E_2_/T ratio was significantly higher in HIV-infected men with primary hypogonadism than in patients with eugonadism or compensated hypogonadism (p<0.05) (Figure 3b). Both BMI and VAT were significantly higher in HIV-infected men with secondary hypogonadism compared with subjects with eugonadism or compensated hypogonadism (p<0.05) (Figures 3c and 3d, Table 4).

Lumbar and femoral BMD did not vary among the four groups (Table 4). Even when patients were grouped according to their t-score into patients with osteoporosis, osteopenia and normal BMD, the percentage of men with impaired BMD was similar in the four groups. The number of months of NNRTI and NRTI exposure was significantly different among the four groups (p<0.05) (Table 4).

### Predictive factors for HIV-related low serum T

Stepwise linear multiple regression analysis for serum total T as the dependent variable was performed in all 1325 HIV-infected men, using serum LH as the forced entry variable, and patients age, body composition parameters, and HIV infection parameters as independent variables generating four different models (Table 7). The main predictor of serum total T resulted VAT, followed by age and BMI (Table 7).

**Table 7:** Stepwise linear multiple regression estimating predictive factors for serum total T in the HIV-infected men.

Dependent variable: Testosterone			
Stepwise linear multiple regression	Variables entering the model		
	β	P level	r^2^
**Model 1**			
LH (forced entry)	+6.947	<0.001	0.032
**Model 2**			
LH (forced entry)	+6.441	<0.001	0.055
VAT	−0.360	<0.001	
**Model 3**			
LH (forced entry)	+6.672	<0.001	0.061
VAT	−0.304	<0.001	
Age	−2.316	0.003	
**Model 4**			
LH (forced entry)	+6.424	<0.001	0.065
VAT	−0.200	0.011	
Age	−2.316	0.003	
BMI	−4.364	0.010	

*β: coefficient of regression. P level: significance of correlation. r^2^: Pearson correlation coefficient. The variables considered into stepwise multiple linear regression are: LH, VAT, age, BMI, waist circumference, hip circumference, SAT, TAT, total body fat at DXA, truncal fat at DXA, total lean mass at DXA, truncal lean mass at DXA, months of HIV-infection, viral load, CD4 count, CD4 nadir.*

## Discussion

This study establishes that low serum total T is a common endocrinological finding (16%) among young/middle-aged HIV-infected men, mainly with lipodystrophy, treated with HAART (Table 2, Figure 2). Similar results are usually observed in non infected men aged 40–69 [4], [5]. However, the latter group exhibit a lower percentage (6%) of androgen deficiency [6], [30] than the HIV-infected men evaluated in this study. Thus, T deficiency seems to be more common in HIV-infected men than in the general population [2], [6], [30]. The absence of a control group is a limitation of the present study, but the prevalence of T deficiency in the general population has been extensively studied [1]–[6], [30] allowing indirect comparison. T deficiency is uncommon before the age of 40 in non infected men [2], unlike the HIV-infected men analyzed in this study. Usually, in non infected men younger than 40 hypogonadism is due to a congenital defect of either the hypothalamic-pituitary axis, an extremely rare condition, or the testes. Acquired hypogonadism (e.g. hypothalamic or pituitary diseases, testicular injuries) is even rarer, such that the prevalence of hypogonadism before the age of 40 is minimal. In fact, Vermeulen & Kaufman [2] found only one subnormal serum T in 105 men aged 20–40 years. The finding of low serum total T in 10.6% of patients aged 30–39 (Table 3) suggests that serum T starts to decline at an early age in HIV-infected men, a conclusion supported by previous studies [7], [12], [13], [15]–[17]. Together with other comorbidities characteristic of HIV infection, the early decline of serum T should be considered an additional element of HIV-related premature aging [31].

The percentage of men having low serum T in the present study is similar to that suggested by other reports [12], [17]. The large sample size investigated by us allows to establish the prevalence of T deficiency in the context of HIV, improving the consistency of the limited, available information obtained in previous studies performed on smaller numbers of subjects (less than 300) [7], [13]–[16] and suffering from the limitations indicated in the introduction.

The novelty of this study lies in the detailed characterization of T deficiency by using serum T in tandem with serum LH levels in a large number of HIV-infected men treated with HAART and mostly lipodystrophic, a comorbidity rarely investigated earlier in small subsets of subjects [12], [14]–[17]. The results of this study show that HIV-infected patients often have normal serum total T, but elevated LH (compensated hypogonadism). This suggests a possible clinical condition that precedes primary gonadal failure. This finding indicates that this category of patients requires close monitoring given their susceptibility to developing overt hypogonadism.

As most of the HIV-infected patients with T deficiency had inappropriately low serum LH (14%), a primary impairment of pituitary gonadotropin secretion could be postulated (Figure 2, Table 4). Thus, the hypothalamic-pituitary axis should be regarded as the main element involved in the development of T deficiency in HIV-infected patients, as previously suggested [11], [12], [16], [22]. Among the factors analyzed, visceral adipose tissue and BMI are found to be predictive of and inversely related to serum total T in HIV-infected men. These results suggest that the amount of VAT may be somewhat involved in suppressing the hypothalamic-pituitary axis and in decreasing serum LH in HIV-infected men, as reported in non infected, obese men [4], [32], [33]. In the absence of overt obesity, fat redistribution due to HIV-related lipodystrophy leads, in fact, to increased visceral adiposity. As estradiol is known to exert a strong inhibitory effect on LH secretion by acting on both the hypothalamus and the pituitary in men [34], the increased serum estradiol levels (Figure 3a) related to adiposity could explain secondary hypogonadism in HIV-infected men [4], [32], [33]. High visceral adiposity (Figure 3c, Figure 3d) and increased T aromatization into estradiol (Figure 3b), in fact, account for increased serum estradiol levels in HIV-infected men with secondary hypogonadism. This information is of clinical relevance since it indicates that T deficiency in HIV-infected patients with visceral adiposity may potentially be reversed through weight loss and visceral fat reduction [4], [32].

The models generated by multiple regression analysis, however, explain only a small percentage of the variance of total serum T, implying that other factors, not assessed in this setting, in addition to adiposity, could contribute to the circulating amount of T in HIV-infected men. Among them, the virus itself and the medications combined in HAART schemes could be implicated in the suppression of the hypothalamic-pituitary-gonadal axis [13], [14], [16], [22], [35]. However, current knowledge in the field remains extremely poor and requires further investigation [22], [35]. Recently, circulating serum T has been suggested to be a marker of illness associated to the general state of health, since low circulating levels of T are often found in men with poor general health [23], [33], [36]. Accordingly, the greater the number of comorbidities, the higher the frequency of T deficiency [33]. Therefore, the hypothesis should be considered that the concomitant presence of several comorbidities [37] and the corresponding worsening of general health, both common in the context of HIV, may have contributed to the high incidence of hypogonadism observed in this study. Accordingly, HAART treatment ensures viral suppression and avoids AIDS development [37] but does not fully restore health [37], [38]. The persistent imbalance of the immune system, the chronic inflammation state and the toxicity of the drugs are responsible for several non AIDS-related complications resembling those occurring during aging in non infected subjects [38]. Their onset at an early age in HIV-infected men supports the hypothesis of premature aging [31], [38]. T deficiency could be included in this process, but presently it is not known whether the premature decline in serum T in HIV-infected men reflects premature aging or if it depends on the health status in terms of concomitant comorbidities and/or frailty. The study design of this study was not specifically addressed at identifying the mechanisms involved in premature aging of the hypothalamic-pituitary-gonadal axis, although we could prove that the gonadal status in young/middle-aged HIV-infected patients resembles that of non infected older men. This process of premature aging in HIV-infected men is known to affect neurocognitive aspects [39], the cardiovascular system [38] and tumorigenesis [40] in a manner otherwise typical of older men [31], [38]. Accordingly, gonadal function may be affected by these multifactorial events (e.g. the presence of several comorbidities, an altered pattern of chronic inflammation, immune system failure and viral replication), which are responsible for the acceleration of the aging process [31], [38]–[40]. Alterations in the immune system and chronic inflammation are known to interfere with the hormonal control at central hypothalamic-pituitary level; similarly, adipokines produced by the adipose tissue may exert much the same effect [32]. We speculate that some of these mechanisms operate in HIV-infected men showing increased visceral fat, alterations in the immune function and a chronically activated inflammation [38].

The lack of the SHBG measurement and, therefore, of free serum T represents a limitation of this study. The use of total serum T alone may have resulted in an underestimation of the prevalence of biochemical hypogonadism since calculated free serum T has been suggested as more appropriate in the context of HIV due to the possible rise in serum SHBG in these patients [17]. Changes of SHBG levels in the HIV context remain, however, controversial, since increases in SHBG with concomitant weight loss, [7], [17], [41], [42], decreases in SHBG [43], and no modifications of serum SHBG [13] have been described. Testing of serum total T has several advantages, in clinical practice, since it is widely available, accurate, unexpensive and reliable [44]. On the other hand, the accuracy of calculated free T has recently been questioned because of the possible sum of errors deriving from both serum total T and SHBG assays [44]–[46]. The measurement of serum T in a single blood sample is another limitation of the present study, since serum T may vary overtime and two different measurements are required in clinical practice to determine biochemical T deficiency with certainty [21], [23]. A single T measurement could possibly result in an overestimation of samples with low serum total T, since it is documented that a second measurement may provide results falling within the normal range [21], [47]. Hence, a single quantification of serum T fairly reflects the amount of circulating T and provides consistent and reliable information if performed on a large sample of patients, as documented in large epidemiological studies [48]. In the present study, the largest and most comprehensive dataset on serum T in the context of HIV, the high number of patients studied counterbalances, at least in part, the tendency to overestimate low serum T when using a single measurement. In addition, the single measurement of serum total T was coupled with the corresponding LH value in each patient enrolled, thus obtaining much complete information on the real gonadal status in these patients. In clinical practice, the confirmation of androgen deficiency by means of a second serum T measurement is a valid procedure, especially prior to starting T replacement treatment in order to avoid unnecessary overtreatment [21], [22]. The present study reveals that signs and symptoms of hypogonadism and the biochemical evidence of hypogonadism can be uncoupled in the context of HIV infection. This results from the concomitance of several confounding factors, such as the high prevalence of sexual problems and the significant bone loss occurring in HIV-infected men, irrespective of the patient's gonadal status [27], [49], [50]. Accordingly, we did not find significant differences in BMD, osteopenia, osteoporosis or in the percentage of impaired IIEF-15 scores between HIV-infected men with or without T deficiency. All these elements result in the overlap of signs and symptoms of androgen deficiency with those related to HIV infection [27], [49], [50]. This result confirms the low specificity of signs and symptoms of androgen deficiency in men with HIV infection [17], [51], suggesting that confounding elements should be considered when diagnosing androgen deficiency in this context [33], as in other chronic illnesses [8], [49]. While, in non infected men, the biochemical evidence of low serum T reaches clinical relevance only in the presence of symptoms of androgen deficiency [30], [33], the clinical diagnosis of T deficiency in HIV is complicated by the low specificity of signs and symptoms of hypogonadism. The latter, in fact, loss their value in confirming the clinical diagnosis of hypogonadism when low serum T is already documented in HIV-infected men.

In conclusion, T deficiency is frequent in young/middle-aged HIV-infected men with lipodystrophy, as are other endocrine diseases in the context of HIV infection [27], [35], [37]. T deficiency seems to occur at a younger age than would be expected among non infected subjects and may be one element of the process of premature or accelerated aging associated with HIV infection [31], [38]. Premature serum T decline is associated with inappropriately low/normal LH and visceral body fat excess, suggesting that weight loss could improve, in some cases, circulating testosterone in HIV-infected patients with increased visceral adiposity. Whether or not HIV-infected men with low serum T are truly hypogonadic and need T replacement remains unclear, but the possible, close relationship between comorbidities, the general state of health and the low serum T as well as the difficulty of diagnosing hypogonadism should not be underestimated. Accordingly, in men with HIV infection and concomitant low serum T, caution is needed in diagnosing true hypogonadism as well as in prescribing T replacement treatment on a large scale . At present, the premature onset of T deficiency in HIV-infected men should be of concern in clinical practice and how to select patients who could benefit from testosterone treatment remains a challenging and a yet unresolved issue, along with the risk of overtreatment and its possible side effects.
